# Revisiting the Self-Assembly of Highly Aromatic Phenylalanine Homopeptides

**DOI:** 10.3390/molecules25246037

**Published:** 2020-12-20

**Authors:** Enric Mayans, Carlos Alemán

**Affiliations:** Departament d’Enginyeria Química and Barcelona Research Center for Multiscale Science and Engineering, Universitat Politècnica de Catalunya, EEBE, C/Eduard Maristany, 10-14, Ed. I2, 08019 Barcelona, Spain; enric.mayans@gmail.com

**Keywords:** molecular engineering, morphological engineering, nanostructures, peptides, staking interactions, supramolecular chemistry

## Abstract

Diphenylalanine peptide (FF), which self-assembles into rigid tubular nanostructures, is a very short core recognition motif in Alzheimer’s disease β-amyloid (Aβ) polypeptide. Moreover, the ability of the phenylalanine (F or Phe)-homopeptides to self-assemble into ordered nanostructures has been proved. Within this context it was shown that the assembly preferences of this family of compounds is altered by capping both the *N*- and C-termini using highly aromatic fluorenyl groups (i.e., fluorenyl-9-methoxycarbonyl and 9-fluorenylmethyl ester, named Fmoc and OFm, respectively). In this article the work performed in the field of the effect of the structure and incubation conditions on the morphology and polymorphism of short (from two to four amino acid residues) Phe-homopeptides is reviewed and accompanied by introducing some new results for completing the comparison. Special attention has been paid to the influence of solvent: co-solvent mixture used to solubilize the peptide, the peptide concentration and, in some cases, the temperature. More specifically, uncapped (FF, FFF, and FFFF), *N*-capped with Fmoc (Fmoc-FF, Fmoc-FFF, and Fmoc-FFFF), C-capped with OFm (FF-OFm), and doubly capped (Fmoc-FF-OFm, Fmoc-FFF-OFm, and Fmoc-FFFF-OFm) Phe-homopeptides have been re-measured. Although many of the experienced assembly conditions have been only revisited as they were previously reported, other experimental conditions have been examined by the first time in this work. In any case, pooling the effect of highly aromatic blocking groups in a single study, using a wide variety of experimental conditions, allows a perspective of how the disappearance of head-to-tail electrostatic interactions and the gradual increase in the amount of π–π stacking interactions, affects the morphology of the assemblies. Future technological applications of Phe-homopeptides can be envisaged by choosing the most appropriate self-assemble structure, defining not only the length of the peptide but also the amount and the position of fluorenyl capping groups.

## 1. Introduction

The formation of amyloid protein deposition is associated with more than 20 human disorders, as for example Alzheimer’s disease, Parkinson’s disease, type II diabetes, and prion disorders [1,2]. The amyloid fibrils, which have a diameter less than 10 nm, are assemblies with a high degree of order along the long axis of the fibril, as has been observed by atomic force microscopy (AFM), transmission electron microscopy (TEM), and scanning electron microscopy (SEM) [3,4,5]. The order of the fibrils is also reflected in their typical green-gold birefringence when examined between cross-polarizers upon staining with the Congo Red dye. In order to understand its implication in previously mentioned disorders and to develop more effective therapies, the molecular mechanism of the process of amyloid fibril formation has been extensively studied using models (polypeptides) and pathologically relevant proteins, even though many points are still unknown [5,6,7,8].

An important finding was that small peptides also form protein-like amyloid fibrils, opening up new avenues to study the mechanism of amyloid formation. For example, the NFGAIL amyloid-forming peptide fragment from the islet amyloid polypeptide (IAPP) showed a structure, molecular conformation and cytotoxicity similar to that of amyloid deposits that are formed by the full-length polypeptide [9]. Moreover, it was found that phenylalanine (Phe or F) plays a key role in the formation of amyloid fibrils [10]. Indeed, the amyloidogenic potential of such aromatic amino acid was proved to be significantly higher than that of the aliphatic ones [11]. These finding together with the ability of these compounds to form well-ordered nanoassemblies that organize in a wide variety of fascinating morphologies, led to the self-assembly study of short Phe-based peptides emerging as a research topic with its own identity.

The pioneering work of Reches and Gazit demonstrated that aromatic diphenylalanine (FF) self-assembles into rigid nanotubular structures and further elucidated its application as scaffolds for casting silver nanowire [12]. Furthermore, the FF sequence was evidenced to play a crucial role in the aggregation of amyloidogenic proteins [13]. After these discoveries, the self-assembly properties of Phe-based short peptides were extensively studied and results were summarized in excellent reviews [14,15,16]. Moreover, different applications have been proposed for those self-assemblies, such as their utilization in drug delivery, flexible electronics and photonics, electrodes for environmental monitoring, and tissue engineering [17,18,19,20,21,22,23]. Interestingly, the mechanism and polymorphism associated with the self-assembly of short peptides have been found to be strongly influenced by the capping groups that are frequently used at the *N*- and/or C-terminus, affecting also to their applications [24,25,26,27,28].

**Scheme 1:**
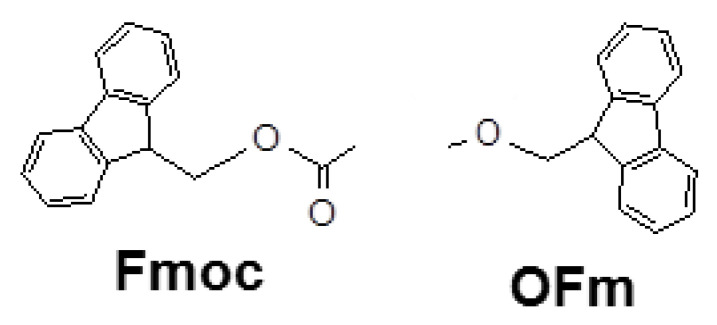
Chemical structures of highly aromatic *N*- and C-capping groups: fluorenyl-9-methoxycarbonyl (Fmoc) and 9-fluorenylmethyl ester (OFm), respectively.

Within this context, in the last years our group has made an effort to understand the influence of highly aromatic capping groups on the morphology of short Phe-homopeptides. More specifically, the assemblies formed by peptides with 2, 3, and 4 Phe residues bearing a fluorenyl capping group at the *N*- and/or C-termini have been studied. Scheme 1 shows the chemical structure of fluorenyl-9-methoxycarbonyl (Fmoc) and 9-fluorenylmethyl ester (OFm) groups, which have been employed as capping groups at the *N*- and C-termini, respectively, in highly aromatic peptides. This work is intended to offer a concise but comprehensive summary of the effects of the number of residues, incubation conditions, peptide concentration, and degree of aromaticity on the morphology and polymorphism of short Phe-homopeptides. For this purpose, previously reported incubation conditions have been re-visited by repeating the experiments and, in addition, some new conditions have been explored to complete the comparison. Only reproducible and frequently detected structures (i.e., representative structures) have been reviewed in the next sections. Furthermore, experimental conditions not studied before (i.e., visited by the first time) are briefly described below and explicitly indicated along the text.

**Figure 1:**
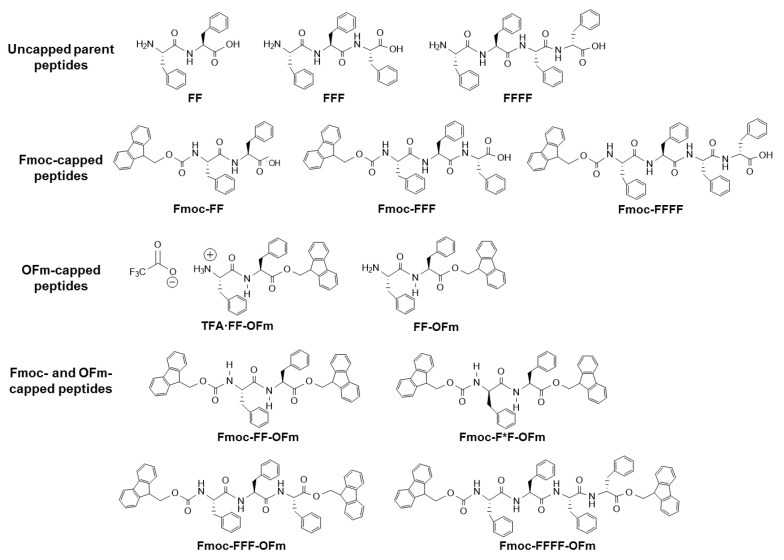
Chemical structures of the peptides examined in this work.

The manuscript is organized as follows. Section 2 briefly describes the experimental conditions used for the preparation of the incubated peptide solutions. After this, the morphologies found for parent Phe-homopeptide assemblies (i.e., uncapped FF, FFF, and FFFF) are discussed in Section 3 for better understanding of the effects induced by Fmoc and OFm capping groups. Section 4 is devoted to the microstructures formed by homopeptides capped at the *N*-terminus with Fmoc (i.e., Fmoc-FF, Fmoc-FFF, and Fmoc-FFFF), while Section 5 describes the morphology of assemblies from homopeptides capped at the C-terminus with OFm (i.e., TFA·FF-OFm and FF-OFm). In the latter section, the utilization of a 50 mM KCl solution to convert TFA·FF-OFm into FF-OFm by eliminating the TFA anion has been examined by first time. Highly aromatic Phe-homopeptides are examined in Section 6 (i.e., Fmoc-FF-OFm, Fmoc-FFF-OFm, and Fmoc-FFFF-OFm), which discusses the effects associated to the simultaneous presence of Fmoc and OFm capping groups. Moreover, such section presents by first time morphologies obtained from water:methanol (MeOH) solutions using moderate and high Fmoc-FF-OFm concentrations. Finally, the conclusions drawn from this comparative study are summarized. Figure 1 displays the chemical structure of all the Phe-homopeptides studied in Section 3, Section 4, Section 5 and Section 6. It should be remarked that the discussion of this work is oriented for understanding the influence of the experimental conditions (i.e., peptide concentration and polarity of the medium) on the morphology of Phe-homopeptides when their aromatic behavior is increased by introducing fluorenyl blocking groups.

## 2. Peptide Solutions

In general, peptide containing solutions (100 μL) were prepared from 5 mg/mL stocks using 1,1,1,3,3,3-hexafluoro-2-propanol (HFIP) as solvent. In some cases, which are explicitly indicated, the HFIP was replaced by *N,N*-dimethylformamide (DMF) for the preparation of the stock solution. The peptide concentration was reduced by adding milli-Q water, MeOH, ethanol (EtOH), isopropanol (^i^PrOH), acetone, or chloroform as co-solvent to the stock solution. More specifically, peptide concentrations of 4, 2, 1, 0.5, 0.25, 0.1, and 0.05 mg/mL were obtained using 4:1, 4:6, 1:4, 1:9, 1:19, 1:49, and 1:99 solvent: co-solvent ratios, respectively. Finally, 10 or 20 μL aliquots were placed on microscope glass coverslips and stored in the lab (at room temperature or at 4 °C) until dryness.

Optical microscopy observations were performed using a Zeiss Axioskop 40 microscope. Micrographs were taken with a Zeiss AxiosCam MRC5 digital camera. Scanning electron microscopy (SEM) studies were performed in a Focused Ion Beam Zeiss Neon 40 scanning electron microscope operating at 5 kV and equipped with an EDX spectroscopy system. Atomic force microscopy (AFM) images were obtained using either a Dimension 3100 Nanoman AFM or a Multimode, both from Veeco (NanoScope IV controller) under ambient conditions in tapping mode.

## 3. Uncapped Phe-Homopeptides

Reches and Gazit discovered the self-assembly of FF in hexagonal nanotubes and microtubes [12]. In this self-assembly process, cyclic hexamers of FF, which are stabilized by head-to-tail NH_3_^+^···^−^OOC interactions, stack forming the nanotube through a network of inter-peptide hydrogen bonds and π–π stacking interactions along the *c*-axis [29]. The hydrophilic core of this nanotube is filled with water molecules that are directly interacting with peptide molecules. The self-assembly of FF can be tuned by the temperature, pH, solvent, peptide concentration in the solution, etc., leading to some morphological diversity (e.g., fibers, wires, tubes, rods, and hydrogels) [30,31,32]. However, the tubular structures, which are the most frequent for FF, are easily obtained in water, organic solvent like HFIP, MeOH, EtOH, acetonitrile, and chloroform, or mixed solutions [30,31,32,33].

**Figure 2:**
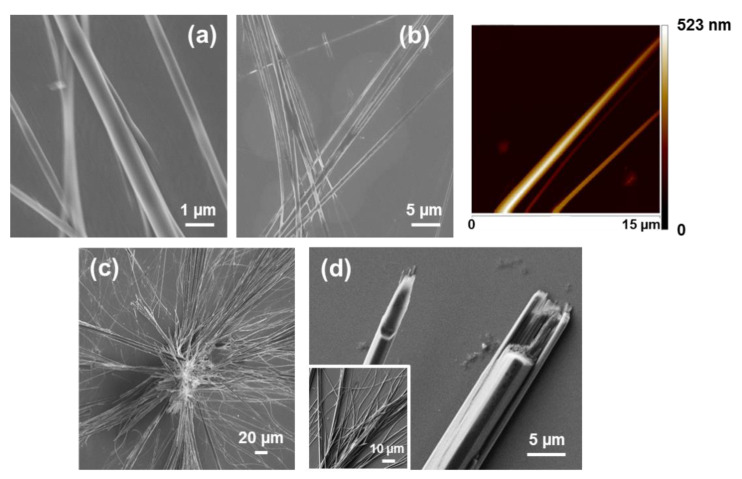
(**a**–**d**) SEM and (**b**, right) AFM images of nano- and microtubular structures obtained from FF solutions in (**a**) 1:49 HFIP:water (0.1 mg/mL), (**b**) 1:99 HFIP:water (0.05 mg/mL), (**c**) 1:4 HFIP:water (1 mg/mL), and (**d**) 1:9 HFIP:EtOH (0.5 mg/mL) mixtures.

Tubular structures obtained from dilute HFIP:water FF solutions (≤0.1 mg/mL) are displayed in Figure 2a,b. When the peptide concentration increases (≥1 mg/mL), the tubes tend to grow forming irregular spherulitic-like nuclei of more than hundred microns of diameter (Figure 2c). Apparently, these structures correspond to dense aggregates of nano- and microtubes that probably share nucleation points among them. Spherulitic structures have been also reported for other amphiphilic Phe-containing dipeptides [34,35]. The definition of FF tubes was found to improve when the water co-solvent was replaced by an organic solvent, such as EtOH [33]. For example, the hexagonal symmetry of FF microtubes can be clearly distinguished in structures obtained in 1:9 HFIP:EtOH (0.5 mg/mL), as is shown in Figure 2d.

**Figure 3:**
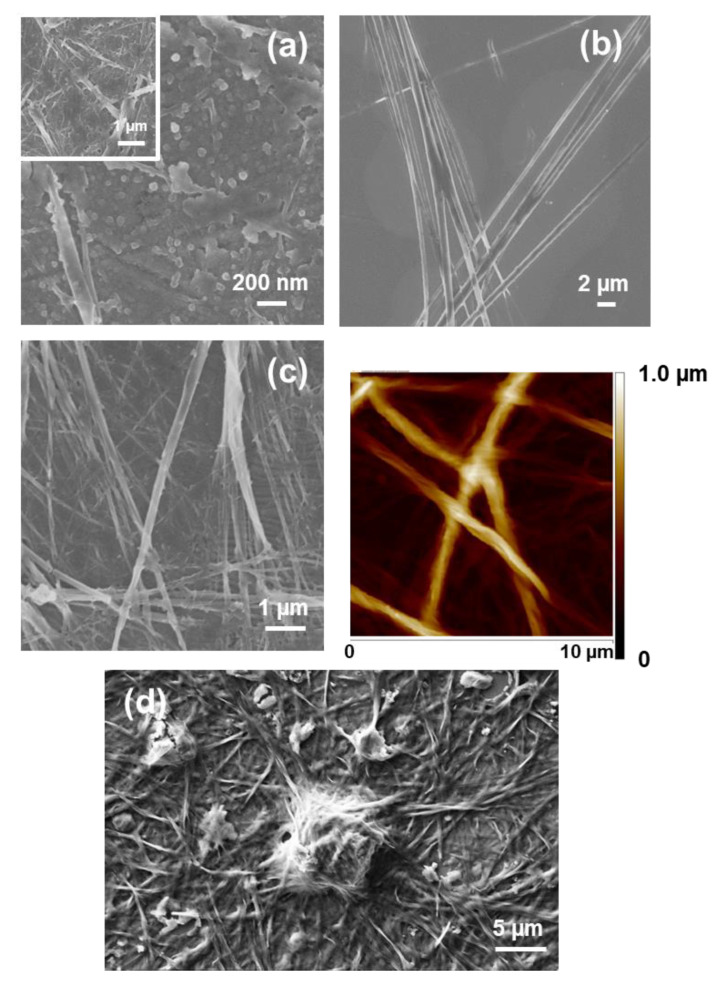
(**a**–**d**) SEM and (**c**, right) AFM images of the structures obtained from FFFF solutions in (**a**) 1:99 HFIP:water (0.05 mg/mL), (**b**) 1:9 (0.5 mg/mL), (**c**) 1:4 (1 mg/mL) HFIP:water, and (**d**) 1:4 (1 mg/mL) HFIP:EtOH mixtures.

Interestingly, uncapped tretraphenylalanine (FFFF) also exhibits a preference towards tubular morphologies. The structures obtained from dilute HFIP:water peptide solutions (0.05 mg/mL) consists on nanospheres (Figure 3a), which appear surrounding incipiently formed fibrous structures. The thickness of the latter structures range from nano-to sub-micrometric, whereas the diameter of the nanospheres is lower than 25 nm. Nano- and microtubular assemblies of micrometric length are clearly stabilized and systematically formed when the peptide concentration is ≥ 0.5 mg/mL (Figure 3b,c). A distinctive characteristic of these elongated structures with respect to those observed for FF is the apparition of defects at the surface. While FF tubes exhibit a smooth surface free of defects (Figure 2), FFFF tends to organize forming multiple superficial knots and branching points.

**Figure 4:**
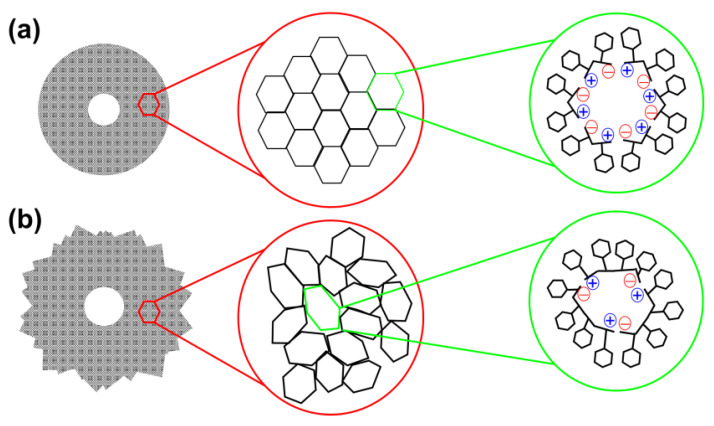
Sketch of the hexagonal and pseudo-hexagonal self-assembly of (**a**) FF and (**b**) FFFF, respectively. The increase of the conformational flexibility and the reduction in the density of head-to-tail electrostatic interactions led to the apparition of surface defects in FFFF.

Although both FF and FFFF contain an even number of Phe residues and, therefore, FFFF is compatible with the hexagonal disposition proposed for FF in tubular structures, theoretical studies evidenced significant differences in the packing of the two peptides [33]. The small size of FF as well the presence of six molecules in the hexagonal packing allowed a regular distribution of the head-to-tail electrostatic interaction. This hexagonal symmetry led to a regular and well-ordered packing, giving place to homogeneous and smooth tubular structures without surface defects, as it is sketched in Figure 4a. Instead, the larger size and, consequently, the higher conformational flexibility of FFFF and the smaller number of molecules involved in the hexagonal self-assembly, with the consequent reduction of head-to-tail NH_3_^+^···^−^OOC interactions (from 6 to 3), led to irregular hexagonal assemblies (Figure 4b). This loss of symmetry was associated not only to the apparition of surface defects but also of branching. Substitution of the water co-solvent by organic solvents also resulted in the formation of microfibers and the apparition of some dense clusters with spherulitic morphology, as is illustrated in Figure 3d for 1:4 HFIP:EtOH (1 mg/mL). This feature supports that FFFF follows the same structural behavior that FF, although with the appearance of numerous defects.

It should be also mentioned that the self-assembly of some FFFF-polymer conjugates have been reported [36,37,38]. More specifically, conjugates with polyethylene glycol (PEG) organize in biphasic structures at high concentrations, which consist in β-sheet FFFF structures and crystallized PEG [36]. The organization of the tetrapeptide and the polymer as independent modules was supported by molecular dynamics simulations [37]. Besides, the structure of FFFF-poly(ethylene oxide) (PEO) conjugates was found to depend on the polymer length. More specifically, nanotubes were observed for short PEO blocks while fibers and worm-like micelles were found for long polymer blocks [38].

**Figure 5:**
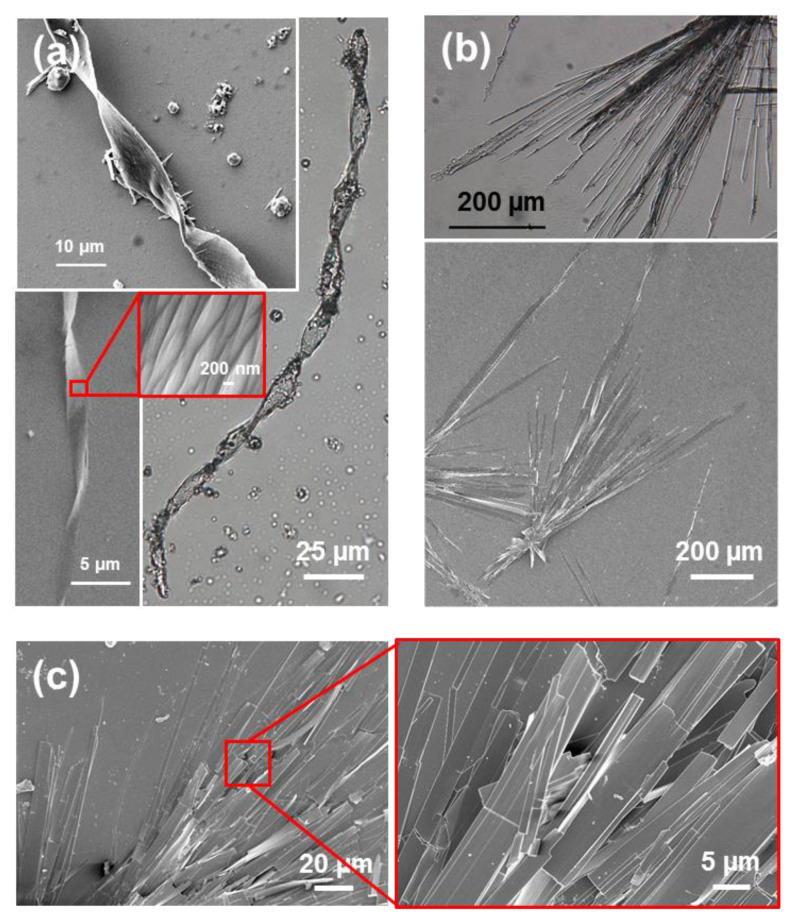
SEM images of the structures obtained from FFF solutions in (**a**) 1:99 HFIP:water (0.05 mg/mL), (**b**) 1:9 HFIP:water (0.5 mg/mL), and (**c**) 4:6 HFIP:water (2 mg/mL).

Completely different self-assemblies with very varied morphologies were observed for FFF, which was attributed to the odd number of Phe residues. More specifically, well-defined microfibrils of ~230 μm with helical ribbons (Figure 5a) were detected for very dilute HFIP:water peptide solutions (0.05 mg/mL) [39]. These structures showed a single twist axis along the long helical axis and a half pitch length of ~50 μm. As is illustrated in Figure 5a, FFF twisted microfibrils are constituted by a dense packing of laminates, which are 320 nm wide, similar to those reported for amyloid fibrils [40,41]. When the amount of water decreases because the FFF concentration increases to 0.5 mg/mL, laminated helical fibrils disappear while well-defined microplates are formed (Figure 5b). These structures are very long in relation with their wideness. The abundance of such microplates increases with the peptide concentration, forming dense stacks of aligned microstructures (Figure 5c). Similarly, Tamamis et al. [42] reported plate-like structures for 2 mg/mL FFF solutions in HFIP:water.

**Figure 6:**
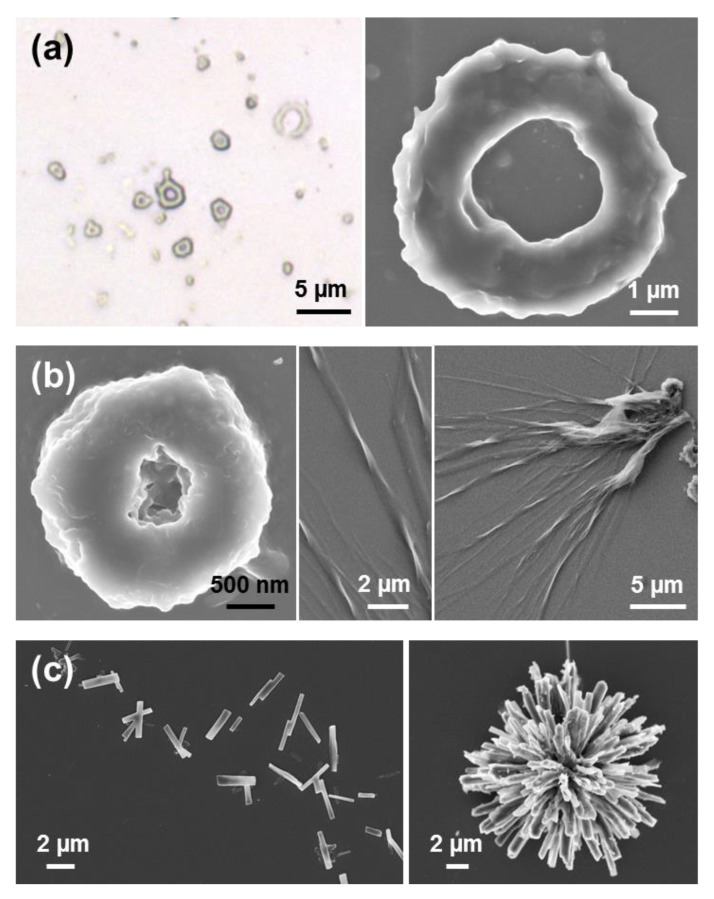
(**a**, left) Optical microscopy and (**a**–**c**) SEM images of the structures obtained from FFF solutions in (**a**) 1:99 HFIP:MeOH (0.05 mg/mL), (**b**) 1:99 HFIP:^i^PrOH (0.05 mg/mL), and (**c**) 1:49 HFIP:^i^PrOH (0.1 mg/mL) mixtures.

Substitution of water by more volatile MeOH in very dilute peptide solutions (0.05 g/mL) results in the formation of well-defined and separated doughnut-shape microstructures of diameter ranging from 2 to 5 µm (Figure 6a) [39]. Moreover, when MeOH is replaced by a less polar solvent, such as ^i^PrOH, doughnut-like structures coexist with twisted microfibers for 1:99 HFIP:^i^PrOH solutions with 0.05 mg/mL peptide concentration (Figure 6b), while aggrupation of microplates even forming supramolecular structures with a flower-like morphology are obtained for peptide concentrations ranging from 0.1 mg/mL (1:49 HFIP:^i^PrOH) to 2 mg/mL (4:6 HFIP:^i^PrOH), as shown in Figure 6c [39].

The size of the toroidal assemblies, which was found to be lower for HFIP:^i^PrOH than for HFIP:MeOH mixtures, regardless of the peptide concentration [39], was associated to the repulsive peptide···solvent interactions. These are expected to decrease with the increasing polarity of the medium. Besides, the formation of the hollow in such assemblies was associated to a solvent evaporation mechanism [39]. Amazingly, similar toroidal microstructures was also described for mixtures of FF and FFF in HFIP:water with a high total peptide concentration (4 mg/mL) [43]. However, in that case the diameter of the structures was attributed to FF···FFF interactions while the formation of the hollow was favored by the competition between FF···water and FFF···water interactions. On the other hand, the formation of flower-shaped aggregates (Figure 6c) was attributed to the balance between attractive intermolecular head-to-tail and π–π interactions and the twisting effect induced by steric hindrance among interlocked elongated microplates [39].

**Figure 7:**
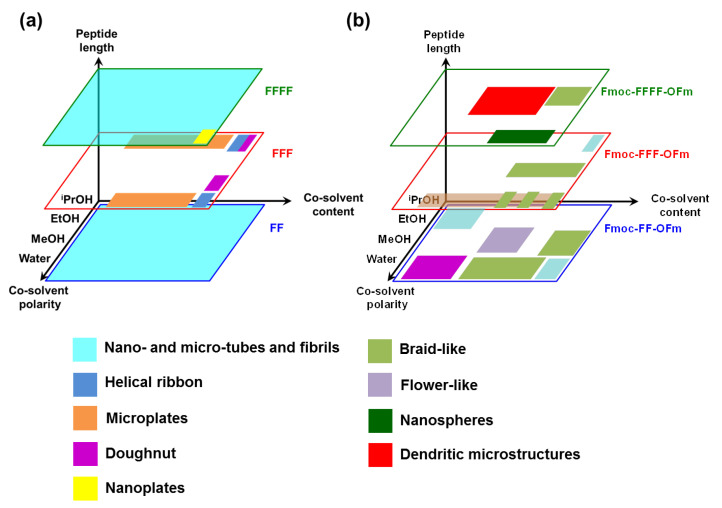
Sketches summarizing the assemblies formed by Phe-homopeptides as a function of the co-solvent, the amount of co-solvent (i.e., the peptide concentration) and the number of Phe residues: (**a**) uncapped and (**b**) capped at both the *N*- and C-terminus with fluorenyl groups.

The self-assembly tendency of uncapped Phe-homopeptides as a function of the peptide length, the polarity of the co-solvent and the amount of co-solvent (i.e., the peptide concentration, which is inversely proportional to the amount of co-solvent), are schematically summarized in Figure 7a. As is shown, tubular and fibrillary structures are the dominant for peptides with an even number of residues, independently of the co-solvent and peptide concentration. Instead, a wide variety of morphologies—which include helical ribbons, individual and grouped microplates, nanoplates, and toroidal structures—have been detected for FFF.

A distinctive characteristic of FFF with respect to FF and FFFF is that some co-solvents and peptide concentrations are not appropriated to form reproducible and well-shaped self-assembled structures. This suggests that intermolecular FFF···FFF interactions define a complex energy landscape with multiple minima, which are very close in energy. Instead, the global minimum of the potential energy hypersurface associated to FF···FF and FFFF···FFFF interactions, which corresponds to hexagonal arrangement that defines the tubular and fibrillary structures (Figure 4), is considerably more attractive than local minima, providing much more stability.

## 4. Phe-Homopeptides Capped at the *N*-Terminus

**Figure 8:**
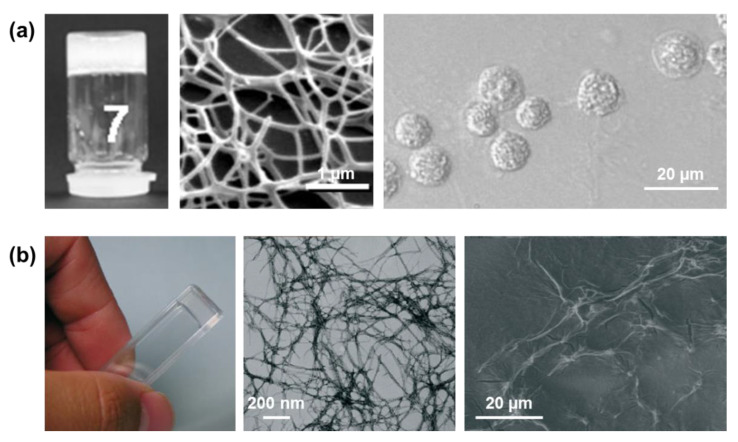
Fmoc-FF hydrogel as simultaneously reported by (**a**) Ulijn and co-workers [44] and (**b**) Gazit and co-workers [45]. (**a**) Glass vial containing the Fmoc-FF hydrogel, labeled as 7 in reference 44 (left), cryogenic SEM image of the hydrogel (middle) and micrographs of cells cultured on the hydrogel (right). (**b**) Photograph of the macroscopic hydrogel (left), TEM image of the fibrous scaffold (middle) and SEM image of the hydrogel (right). Adapted with permission from references [44,45].

Two research groups simultaneously reported that the FF dipeptide bearing the Fmoc group at the *N*-terminus (Fmoc-FF) form stable supramolecular hydrogels due to the formation of supramolecular nanofiber networks (Figure 8), which were utilized as scaffold materials for cell cultures [44,45]. Fibrous networks were detected in a variety of solvents, including non-polar ones, at high peptide concentrations (15 mg/mL). However, macroscopic crystal aggregates were detected in certain solvent systems, like methanol (300 mg/mL) and ethanol (15 mg/mL) [46]. Moreover, the combination of this short hydrogelator with other compounds—such as polysacchararides, polymers, and peptides—has produced novel materials with enhanced properties, stability, and biocompatibility, bringing broad ranges of applications in both the biomedical and industrial fields, as was recently reviewed Accardo and co-workers [47].

**Figure 9:**
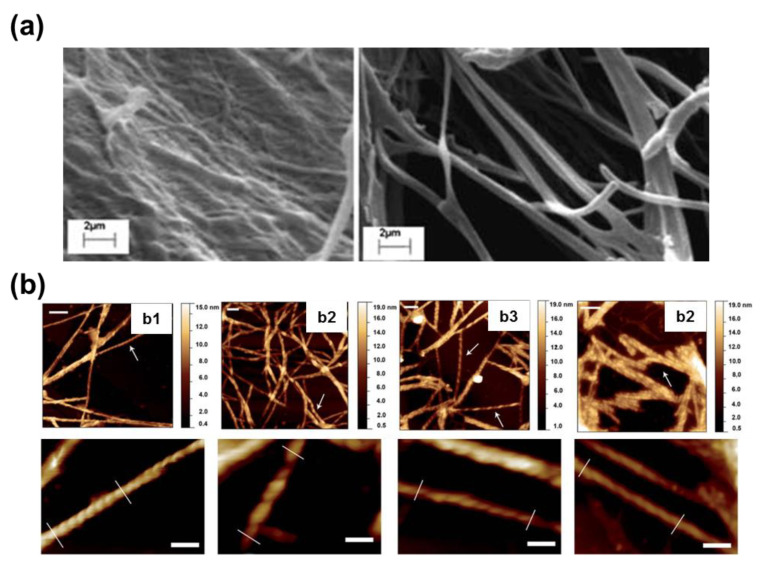
(**a**) SEM micrographs of dried Fmoc-FFF hydrogels obtained at moderate peptide concentration (1.36 mg/mL). Taken with permission from [48]. (**b**) AFM height images (scale bar: 100 nm) of Fmoc-FFF made of all-L (b1), all-D (b2), LDD (b3) and DLL (b4) amino acids. Arrows indicate fibers where twisting is clearly visible. Magnified AFM height images of the twisted fibers are shown below (scale bar: 50 nm). Adapted with permission from [50].

The self-assembly of Fmoc-FFF was explored by Palocci and coworkers [48,49]. These authors used an enzymatic reaction with a lipase for linking Fmoc-F to FF, forming the desired amphiphilic tripeptide under physiological conditions. This bioconversion resulted in a 3D, stable and self-supported Fmoc-FFF hydrogel in only 10 min, which was formed by entangled fibers (Figure 9a) [48]. This enzyme-mediated strategy, which promotes both the self-assembly and gelation of the resulting Fmoc-FFF in presence of cells, was proposed as a good approximation in tissue engineering applications for the direct 3D incorporation of medium and cells through the matrix. Moreover, the Fmoc-FFF hydrogel, which exhibited mechanical properties comparable to natural biopolymers, was used as a scaffold, inducing a significant cell proliferation and increasing the production of the neurotrophic factor NGF [49]. The same strategy was used to construct Fmoc-FFF made of all-D-amino acids or alternating DLL and LDD amino acids [50], self-assembled hydrogels made of complex networks of interconnected nanofibers being formed in all cases (Figure 9b). The D-amino acid-containing hydrogels may be able to resist protease degradation and, therefore, could be considered as promising biomaterials for in vivo tissue engineering and sustained drug release applications.

**Figure 10:**
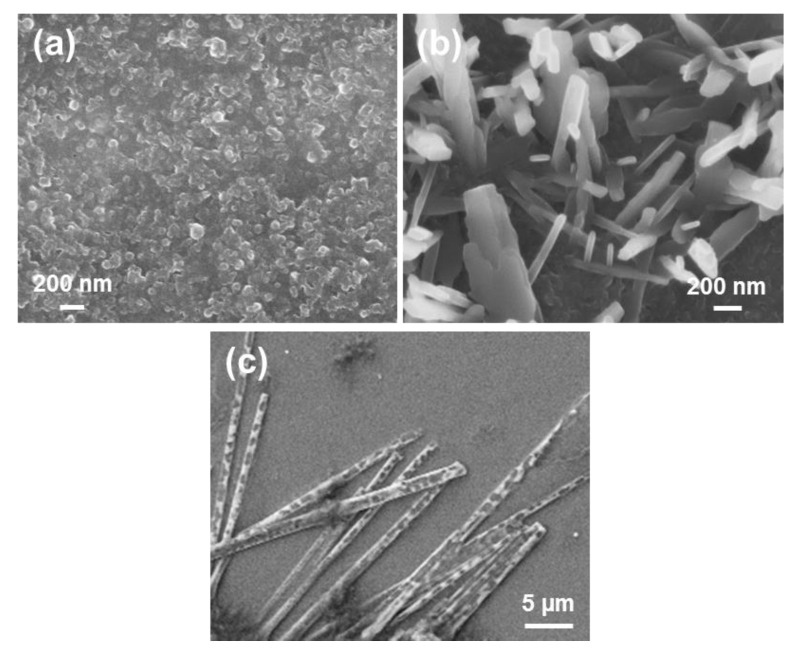
SEM micrographs of the structures obtained from Fmoc-FFFF solutions in (**a**) 1:49 HFIP:water (0.1 mg/mL), (**b**) 1:9 HFIP:water (0.5 mg/mL), and (**c**) 3:47 HFIP:EtOH (0.3 mg/mL).

Fmoc-FFFF was found to assemble in structures completely different from those found for FF and FFFF [33]. This was attributed to the fact that Fmoc-FFFF prefers a parallel β-sheet arrangement rather than an antiparallel one, favoring the formation of π–π stacking interactions between neighboring Fmoc rings (i.e., Fmoc···Fmoc) and between phenyl rings of adjacent peptides (i.e., Phe···Phe). Thus, π–π stacking interactions are strong enough to compensate other polar interactions, which are predominant in the Fmoc-capped homopeptides with a lower number of Phe residues. As consequence, Fmoc-FFFF forms small nanospheres in dilute HFIP:water peptide solution (Figure 10a), while microaggregates of nanoplates emerge from such layers of nanospheres when the peptide concentration increases to 0.5 mg/mL (Figure 10b). The latter nanostructures are micrometric in length, submicrometric in width and nanometric in thickness. However, other assemblies appear when the water was replaced by a less polar and volatile organic solvent. This feature is illustrated in Figure 10c, which shows microfibrils obtained using HFIP:EtOH solutions at moderate peptide concentration (i.e., 0.3 mg/mL).

It is worth noting that, although fibrillary structures were identified in Fmoc-FFF [48,49,50] and Fmoc-FF [51,52,53] hydrogels, easily formed nanoplates and submicrometric star-like aggregates were not reported for such *N*-Fmoc-protected analogues. Theoretical calculations at the DFT level indicated that the interactions between aromatic rings are the driving forces responsible for the unique Fmoc-FFFF assemblies [33], whereas the contribution of the π–π stacking is not the predominant in Fmoc-FF and Fmoc-FFF.

## 5. Phe-Homopeptides Capped at the C-Terminus

**Figure 11:**
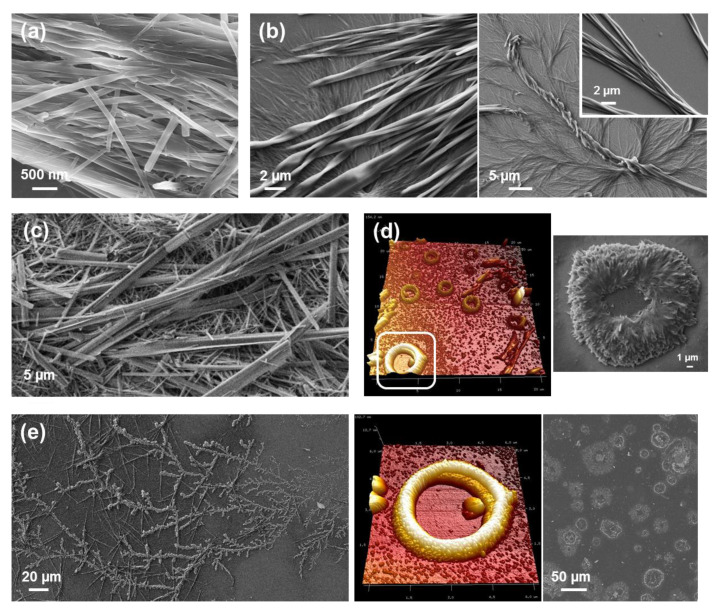
SEM micrographs of the structures obtained at 4 °C from TFA·FF-OFm solutions in (**a**) 4:6 HFIP:water (2 mg/mL) and (**b**,**c**) 1:9 HFIP:water (0.5 mg/mL) mixtures. The solution deposited in the coverslips was fresh in (**b**) while it was previously stored for 12 days at room temperature in (**c**). (**d**) AFM images (20 × 20 and 6 × 6 μm^2^ at the top and the bottom, respectively) and (**d**,**e**) SEM images of the structures obtained at room temperature from TFA·FF-OFm solutions in (**d**) 4:1 HFIP:water (50 mM KCl) (4 mg/mL) and (**e**) 1:9 HFIP:water (50 mM KCl) (0.5 mg/mL).

To the best of our knowledge, the self-assembly of Phe-homopeptides capped with a fluorenyl group at the C-terminus have been only studied for TFA·FF-OFm, where TFA refers to trifluoroacetate anion, and FF-OFm [54]. The main differences between Fmoc-FF and TFA·FF-OFm dipeptides are: (a) the position of the capping fluorenyl group; and (b) the stabilization of the free amino end group through the TFA anion, which exerts a charge shielding effect and reduces the strength of electrostatic intermolecular interactions. These differences provide significant morphological versatility, especially when the self-assembly is driven at low temperature (4 °C) by a slow solvent evaporation process [54]. For example, assemblies from HFIP:water solutions with relatively high peptide concentrations (2 mg/mL) consist on bundles of entangled nanofibers with a diameter of ~100 nm (Figure 11a). When the peptide concentration decreases to 0.5 mg/mL by increasing the amount of water co-solvent and the self-assembly process is maintained at 4 °C, strands intertwine forming helical ribbons (Figure 11b), which in turn were found to intertwine giving amyloid fibrils [54]. This capacity to produce amyloid fibrils should be attributed to the OFm capping group since previous studies on TFA·FF-OBzl, where OBzl refers to the C-terminus benzyl ester capping group, evidenced assemblies of randomly oriented fibers, while no twisted ribbon of amyloid fibril was detected [55]. Overall, such observations suggested that π–π stacking interactions involving the highly aromatic fluorenyl ring were playing an important role in the nucleation and growing of amyloid fibrils [54]. This hypothesis was later confirmed by theoretical calculations on molecular models constructed by packing pre-assembled β-sheets [56].

Nanofibers transform into hierarchically assembled long plates (Figure 11c) when the kinetic control provided by the slow evaporation of the solvents at 4 °C and the increase of water co-solvent by decreasing the peptide concentration, is combined with a thermodynamic control step. This was achieved by storing for 12 days the TFA·FF-OFm solution in 1:9 HFIP:water at room temperature, before placing at 4 °C until dryness. It is worth noting that although the structures displayed in Figure 11b,c were obtained using 1:9 HFIP:water peptide solutions (0.5 mg/mL), twisted fibers were completely absent in Figure 11c, reflecting the importance of the incubation conditions on the kinetic and thermodynamic control of the self-assembly process.

Elimination of the stabilizing TFA anion by neutralizing with 5% NaHCO_3_, which resulted in the FF-OFm dipeptide, produced fibrous morphologies (not shown) when solutions with a peptide concentration of 2 and 0.5 mg/mL, (i.e., 4:6 and 1:9 HFIP:water (5% NaHCO_3_), respectively) were incubated until dryness at 4 °C [54]. Although the size of the fibers decreased with the peptide concentration (i.e., micro- and nanofibers were obtained for 2 and 0.5 mg/mL peptide solutions, respectively), figures associated to twisted ribbons and amyloid fibrils were not detected in any case.

In the present work, we have explored by first time the utilization of a 50 mM KCl solution to eliminate the TFA anion since, due to its small size, the Cl^−^ anion is expected to interfere less than the carbonate in the self-assembly processes. Interestingly, HFIP:water (50 mM KCl) solutions with high peptide concentrations (4 mg/mL) produced abundant and well-defined toroidal microstructures at room temperature (Figure 11d). The doughnut-shape morphology of these structures resembles that observed for uncapped FFF from HFIP:MeOH and HFIP:^i^PrOH mixtures with dilute peptide concentration (Figure 6a,b). However, the diameter of the toroid is small and homogeneous for FFF (i.e., 2–5 µm), while it ranges from 5 to almost 65 µm for FF-OFm. This dimensional variability has been associated to the high peptide concentration in the neutralized peptide solution. Instead, branched dendritic structures were formed when the peptide concentration decreased to 0.5 mg/mL in 1:9 HFIP:water (50 mM KCl) mixtures (Figure 11e). Although all these microstructures are probably influenced by the salt used to neutralize TFA·FF-OFm, comparison of these results with those reported Fmoc-FF reflects the important role of highly aromatic capping groups.

**Figure 12:**
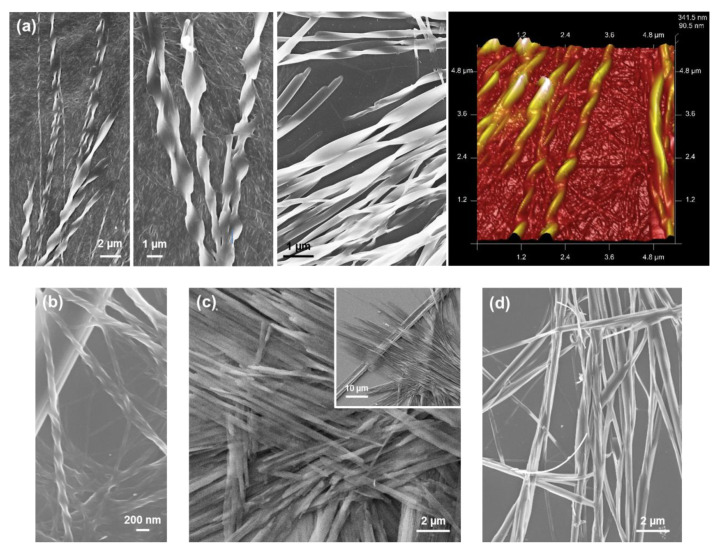
(**a**–**d**) SEM micrographs and (**a**) AFM image (5.4 × 5.4 µm^2^) of the structures obtained at room temperature from TFA·FF-OFm solutions in (**a**) 1:4 HFIP:MeOH, (**b**) 1:4 DMF:MeOH, (**c**) 1:4 HFIP:chloroform mixtures (1 mg/mL in all cases), and (**d**) pure HFIP (5 mg/mL).

Twisted amyloid fibrils similar to those achieved from TFA·FF-OFm dilute HFIP/water solutions were also reported for using organic co-solvents [56]. Indeed, the characteristics of the fibrils were regulated through the properties not only of the co-solvent but also of the organic solvent used to prepare the stock solution. This is illustrated in Figure 12a,b, which display fibrils obtained using 1:4 HFIP:MeOH and 1:4 DMF:MeOH (1 mg/mL in both cases). As it can be seen, important structural parameters, such as the diameter (~0.8 µm and 150 nm, respectively) and density of fibrils, were defined by the solvent used to prepare the stock peptide solution.

However, characteristics of the organic co-solvent also play an important role, as is evidenced in Figure 12c,d. Replacement of MeOH by a more volatile co-solvent, like chloroform, resulted in the formation of flat non-twisted structures of micrometric length similar to long nanoplates (Figure 12c), whereas complete elimination of the co-solvent yielded branched microfibers with abundant superficial defects (Figure 12d). Overall, the polarity of the organic mixture controls the dimensions and density of TFA·FF-OFm fibrils. Thus, the drying process, which obviously depends on the properties of the solvent and co-solvent (e.g., the vapor pressure, the dielectric constant and the existence of hydrogen bonding groups), was found to play a decisive role in the formation of TFA·FF-OFm amyloid-like fibrils [56].

## 6. Phe-Homopeptides Capped at both the *N*- and C-Terminus

**Figure 13:**
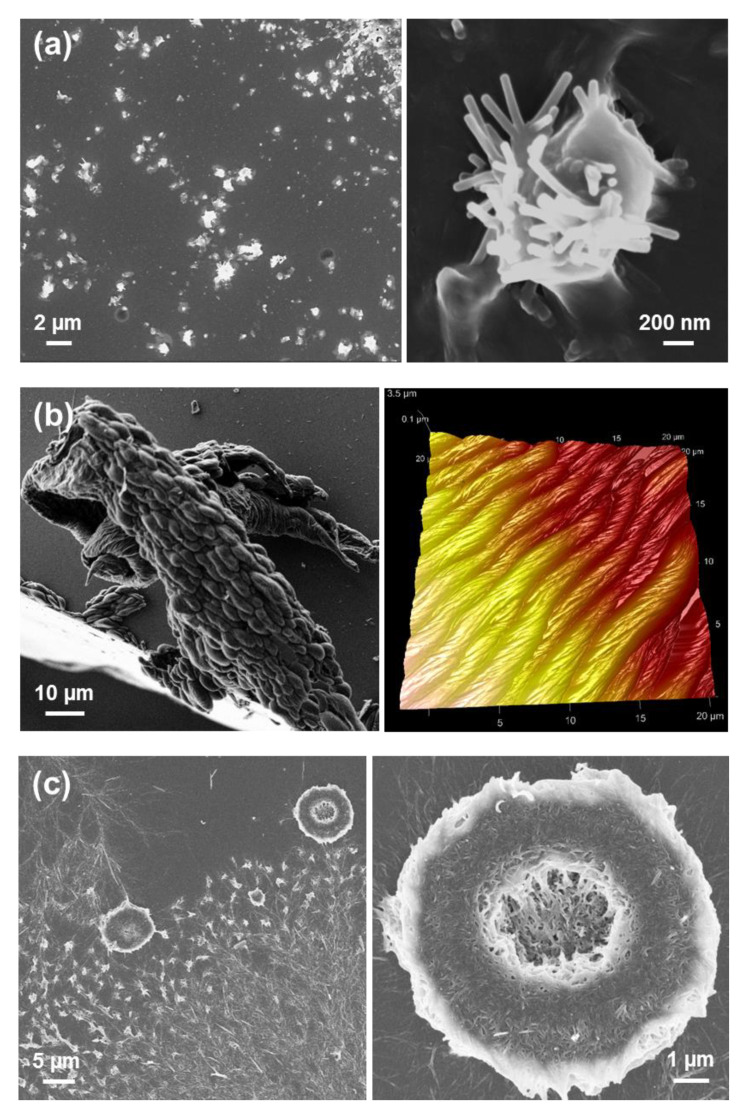
(**a**–**c**) SEM and (b) AFM (20 × 20 µm^2^) images of the structures obtained from Fmoc-FF-OFm solutions in (**a**) 1:99 HFIP:water (0.05 mg/mL), (**b**) 1:9 HFIP:water (0.5 mg/mL), and (**c**) 4:1 HFIP:water (4 mg/mL) mixtures.

The self-assembly of Phe-homopeptides capped at the *N*- and C-terminus by Fmoc and OFm groups, respectively, was studied in recent work [57]. The combination of such two aromatic capping groups, together with the contribution of the phenyl side groups of the Phe residues, promoted the formation of π–π stacking and hydrophobic interactions, resulting in a relatively rapid self-assembly kinetics. Fmoc-FF-OFm forms distributions of small microaggregates (1–1.5 µm) constituted by nanofibers with a diameter of ~50 nm and submicrometric length (Figure 13a) when the content of water is very high (1:99 HFIF:water) and, therefore, the peptide concentration is very low (0.05 mg/mL). Instead, the peptide assembles in stacked braid-like microstructures at the edges of the coverslips (Figure 13b) when the peptide concentration increases and, therefore, the amount of water in the solution decreases. These structures are observed for peptide concentrations ranging from 0.5 to 2 mg/mL, even though the size and degree of compactness of the braids decreases with the peptide concentration in the solution [57]. Similar stacked braid-like assemblies were obtained in HFIP:MeOH mixtures with a high content of co-solvent (i.e., peptide concentrations ranging from 0.05 to 0.1 mg/mL) [57].

When the content of water in the peptide solution is low, hollow toroidal microstructures with diameters comprised between 4 and 10 µm are observed (Figure 13c). These structures resemble those obtained for FF-OFm using a similar peptide concentration (4 mg/mL in Figure 11d) and uncapped FFF using very dilute peptide solutions in two organic solvents (Figure 6a,b). The mechanism proposed to explain the formation of these Fmoc-FF-OFm doughnut-like microstructures formed from Fmoc-FF-OFm was the collapse of small gelatinous rounded aggregates with irregular micrometric assemblies [57]. Thus, such collapse was hypothesized to result in the nucleation center and subsequent growing of the doughnut-like microstructures, which occurred through the diffusion of the peptide molecules located at the central region to the external region. This mechanism was supported by the role played by aromatic capping groups as facilitators of gelation [58].

**Figure 14:**
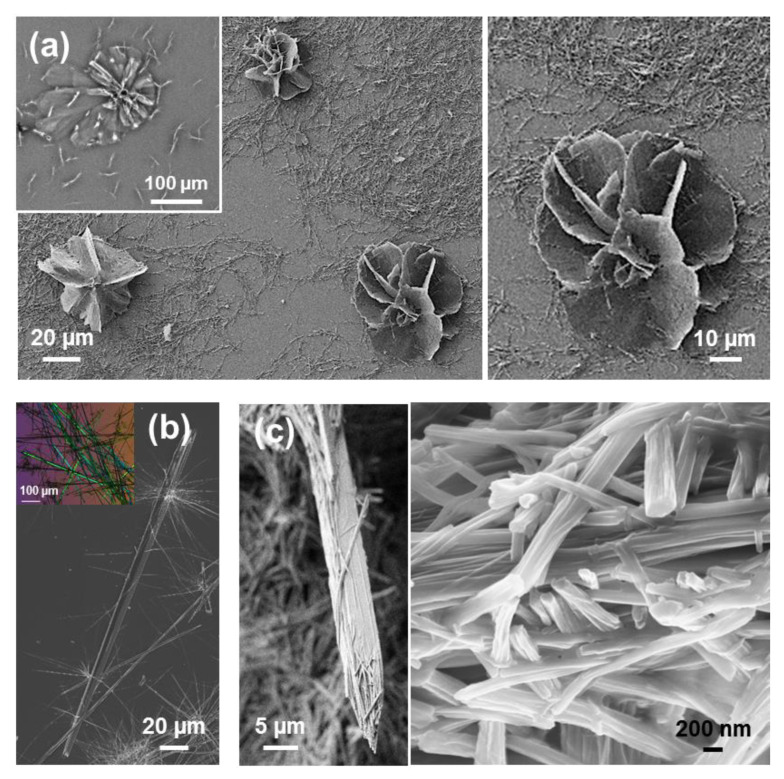
SEM and optical (inset in **b**) micrographs of the structures obtained from Fmoc-FF-OFm solutions in (**a**) 1:4 HFIP:MeOH (1 mg/mL), (**b**) 4:1 HFIP:^i^PrOH (4 mg/mL), and (**c**) 4:1 HFIP:acetone (4 mg/mL) mixtures.

In the present work we have investigated by first time the substitution of water by MeOH at higher peptide concentrations. Interestingly, we have repetitively found the apparition of flower-like microstructures that can be described as ultra-thin microplates growing from close, or even the same, nucleating points (Figure 14a). The diameter of these microstructures, which are similar in shape to the well-known desert roses and appear when the peptide concentration is 1–2 mg/mL, is comprised between 50 and 100 µm. Birefringent microtubes with smooth and regular surface, like those displayed in Figure 14b, are formed rom 4:1 HFIP:^i^PrOH. Although the diameter of these structures presents a large variability, ranging from 1 to 10 µm, the larger ones resemble the hexagonal microtubes observed by Möhwald and co-workers [59] for uncapped FF using 1 mg/mL HFIP:water solutions. Those authors found that FF microtubes were built hierarchically from the growth and confined organization of nanotubes, which in turn were formed by self-assembled FF molecules hexagonally packed through electrostatic head-to-tail interactions (Figure 3a) [59]. Considering that Fmoc-FF-OFm cannot form a hexagonal packing stabilized by electrostatic head-to-tail interactions and discarding the hydrophobic collapse in a 4:1 HFIP:^i^PrOH solution, the assembly of highly aromatic Fmoc-FF-OFm molecules in microtubes (Figure 14b) is probably driven by π–π stacking interactions. Theoretical calculations supported the key role of π–π stacking interactions in the assembly of highly aromatic double capped Phe-homopeptide strands [57]. More specifically, such interactions were found to promote the formation of antiparallel β-sheet assemblies, whereas parallel β-sheets were observed for the corresponding uncapped Phe-homopeptides.

On the other hand, microtubes that clearly come from the self-assembly of Fmoc-FF-OFm nanotubes were obtained from 4:1 HFIP:acetone solutions (Figure 14c). These bundle arrays of nanotubes, which exhibit a diameter comprised between a few tenths of nm and 200 nm, are confined and show a preferential orientation along the longitudinal axis of the microtube. The observation of this hierarchical structure in a water-free environment and the impossibility of Fmoc-FF-OFm to form electrostatic interactions supported the role of π–π stacking interactions in tubular assemblies of uncapped FF was underestimated.

**Figure 15:**
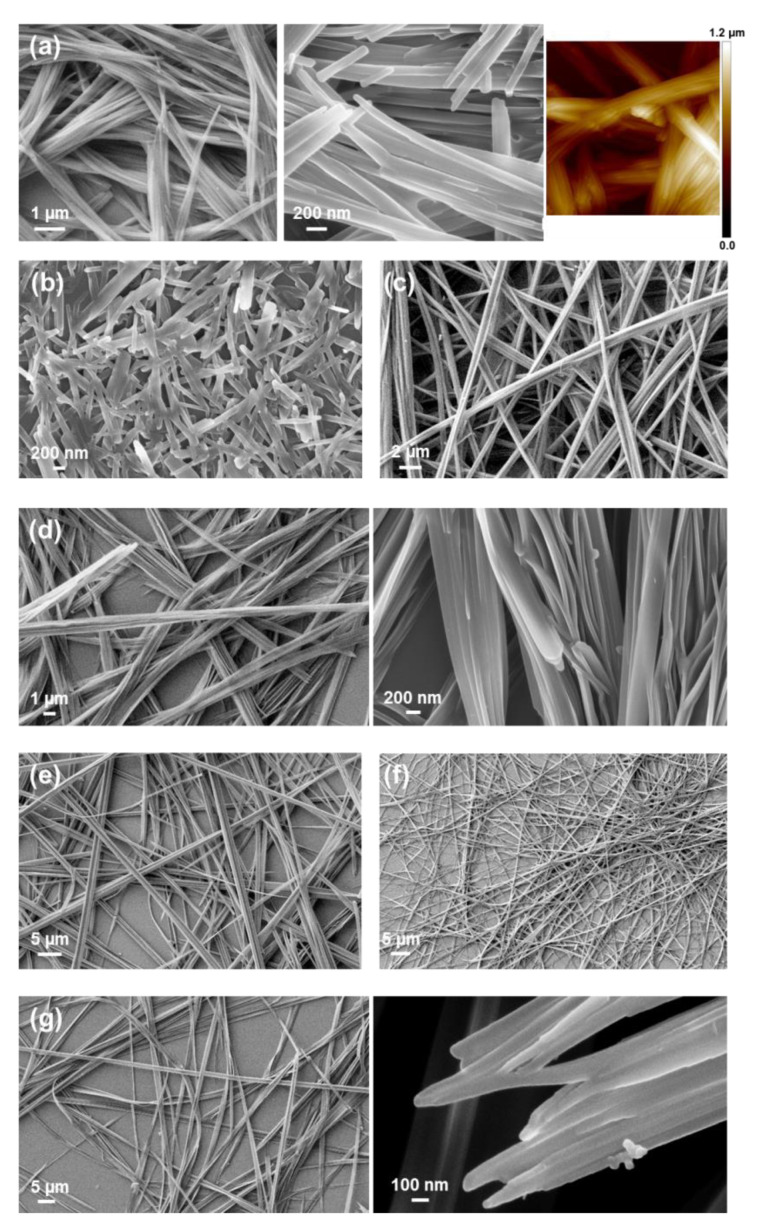
(**a**–**g**) SEM and (**a**) AFM (7 × 7 µm^2^) images of the structures obtained from Fmoc-F*F-OFm solutions in (**a**) 4:6 HFIP:water (2 mg/mL), (**b**) 1:9 HFIP:water (0.5 mg/mL), (**c**) 4:1 HFIP:water (4 mg/mL), (**d**) 4:6 HFIP:MeOH (2 mg/mL), (**e**) 4:6 HFIP:^i^PrOH (2 mg/mL), (**f**) 1:19 HFIP:^i^PrOH (0.25 mg/mL), and (**g**) 4:6 HFIP:acetone (2 mg/mL) mixtures.

The influence of the stereochemistry in the self-assembly of Fmoc-FF-OFm was recently examined [60]. More specifically, the first *l*-Phe residue of Fmoc-FF-OFm was replaced by *d*-Phe and the assembly of the resulting peptide, Fmoc-*d*-Phe-*l*-Phe-OFm (Fmoc-F*F-OFm in Figure 1), was investigated in HFIP:water and HFIP:alcohol mixtures. Results showed that the replacement of *l*-Phe by *d*-Phe restricted the morphological diversity shown in Figure 13 and Figure 14 for the homochiral dipeptide. Fmoc-F*F-OFm exhibited a great propensity to form micro-/nanofibers, independently of the co-solvent and peptide concentration. This is illustrated in Figure 15a–c, which compares the fibers obtained from HFIP:water solutions with different ratios. The diameter of the fibers obtained for a peptide concentration of 2 mg/mL (Figure 15a) decreases and increases when the concentration changes to 0.5 and 4 mg/mL, respectively (Figure 15b,c).

The propensity of the heterochiral Fmoc-F*F-OFm to assemble forming fibers is preserved not only with less polar co-solvents, such as MeOH (Figure 15d) and ^i^PrOH (Figure 15e,f), but also with co-solvents that cannot act as hydrogen bond donors and are much more volatile, such as acetone (Figure 15g). Theoretical studies showed that the formation of Fmoc···OFm aromatic cores is the driving force of the aggregation process of both Fmoc-FF-OFm and Fmoc-F*F-OFm, leading to a decrease in the entropy of the system [60]. However, the preference for antiparallel β-sheet formation was found to be significantly higher for the heterochiral dipeptide than for the homochiral one [60], explaining the singular behavior observed for Fmoc-F*F-OFm in comparison to Fmoc-FF-OFm (i.e., higher tendency to self-assembly and much more restricted structural diversity).

**Figure 16:**
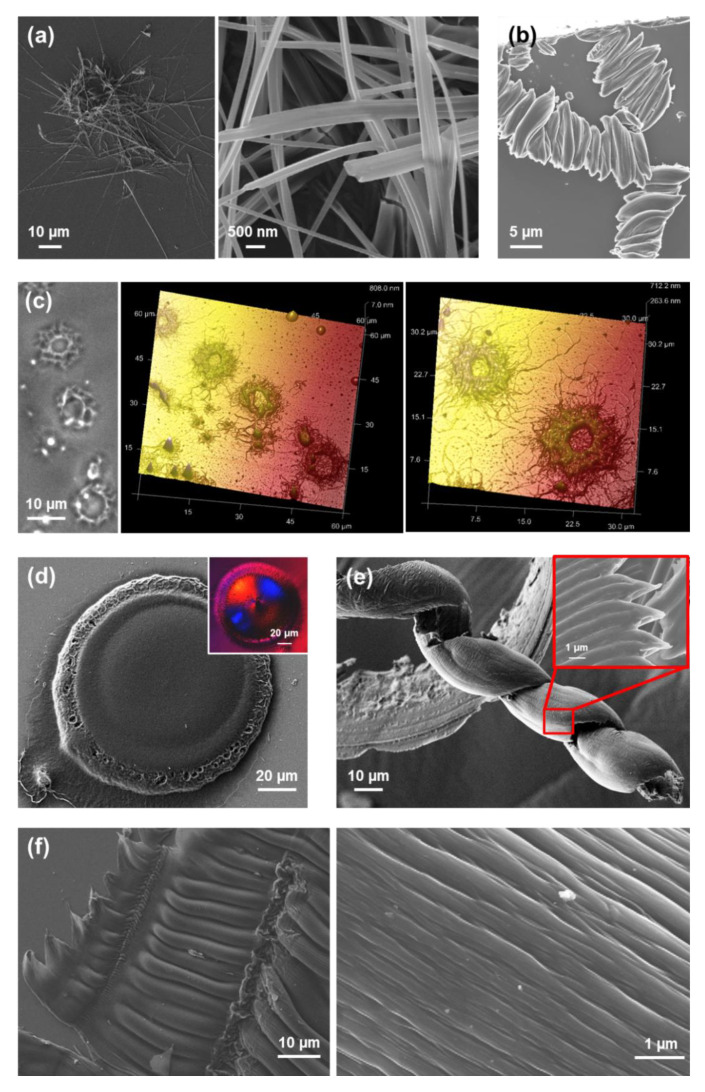
(**a**,**b**,**d**,**e**) SEM, (**c**,**d**) optical and (**c**) AFM (left: 60 × 60 µm^2^; right: 30 × 30 µm^2^) images of the structures obtained from Fmoc-FFF-OFm solutions in (**a**) 1:99 HFIP:water (0.05 mg/mL), (**b**) 1:4 HFIP:water (1 mg/mL), (**c**) 4:1 HFIP:water (4 mg/mL), (**d**) HFIP (5 mg/mL), (**e**) 4:6 HFIP:EtOH (2 mg/mL), and (**f**) 1:9 HFIP:^i^PrOH (0.5 mg/mL) mixtures.

Fmoc-FFF-OFm forms fibers of micrometric length and nanometric diameters (Figure 16a) in very dilute HFIF:water solutions (0.05 mg/mL). These structures coexist with assemblies of stacked braids, which are formed at the edges of the cover glass for peptide concentrations ranging from 0.05 to ~2 mg/mL (Figure 16b). The groups of fibers found at low peptide concentrations transform in well-defined hollow spherulites when the peptide concentration increases up to 4 mg/mL (Figure 16c). Moreover, complete elimination of the co-solvent results in the formation of dense and compact birefringent spherulites (Figure 16d). These disc-like assemblies, which can be described as a very dense tissue of nanofibers, were only detected from 5 mg/mL Fmoc-FFF-OFm solutions in HFIP. This feature reflects a water-induced hierarchical assembly transition from dense nanofibrous spherulitic discs (Figure 16d) to groups of nanofibers (Figure 16a) passing through hollow fibrous spherulites (Figure 16c). It should be mentioned that birefringent spherulitic assemblies were previously reported for hydrophobic Fmoc-SF-OMe [34].

On the other hand, stacked braids similar to those obtained using HFIP:water solutions were also formed from HFIP:EtOH mixtures within the same interval of peptide concentrations. However, when the peptide concentration was comprised between 1–2 mg/mL, the stacking of the braids grew forming 3D hierarchical assemblies with corkscrew-like shapes (Figure 16e). The formation of such 3D structures, which was not observed in assemblies coming from HFIP:water solutions, was attributed to the volatility of EtOH [57]. However, no spherulitic structure was observed in assemblies formed from HFIP:EtOH solutions. On the other hand, HFIP:^i^PrOH solutions were not found to favor the formation of reproducible assemblies for Fmoc-FFF-OFm concentrations higher than 0.5 mg/mL. However, fused submicrometric fibers resembling stacked braids were observed below such threshold concentration (Figure 16f).

The effect of the substrate in the self-assembly of Fmoc-FFF-OFm was reported in a recent work by comparing the assemblies obtained on the surface of silanized and scratched glasses, stainless steel, exfoliate mica, silicon wafer, and different plastic substrates [61]. It was found that the competition between peptide···peptide and peptide···substrate interactions, which depend on the peptide concentration in the solution and the physical properties of the substrate’s surface (i.e., hydrophilicity and roughness), gave place to very different hierarchical organizations—such as stacked braids, spherical, dendritic-like, and jellyfish-like microstructures—among others. Indeed, the surface-mediated polymorphism observed for Fmoc-FFF-OFm was much more noticeable than that reported for uncapped FF. The latter organized forming nanotubular assemblies on glass, silicon, aluminum, and mica, even though the different surface properties of such substrates influenced the density, distribution, and size of the nanotubes [62].

**Figure 17:**
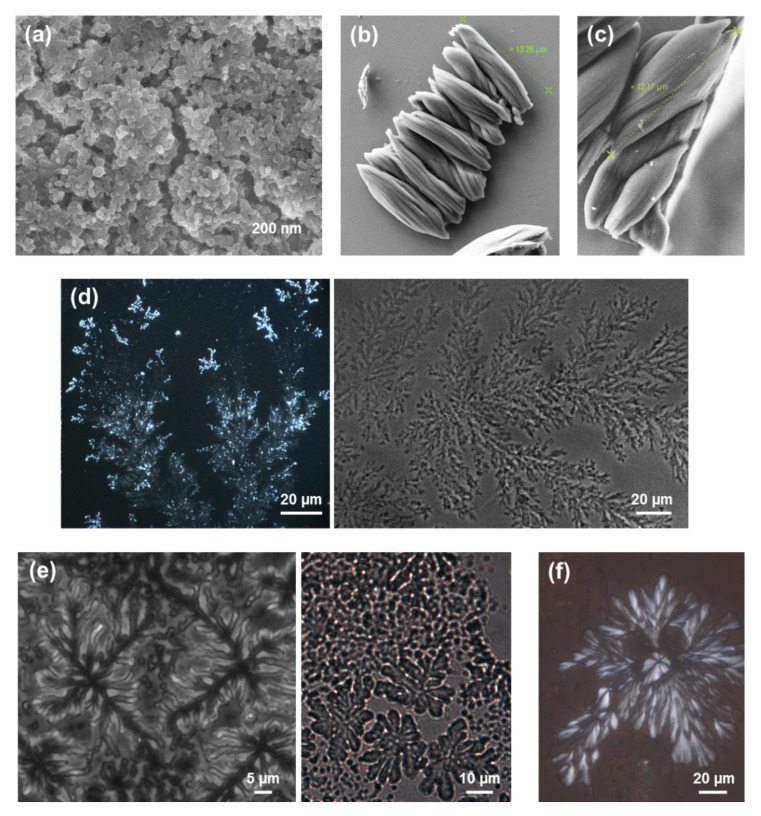
(**a**–**c**) SEM and (**d**–**f**) optical micrographs of the structures obtained from Fmoc-FFFF-OFm solutions in (**a**) 1:9 HFIP:water (0.5 mg/mL), (**b**) 1:9 HFIP:EtOH (0.5 mg/mL), (**c**) 1:99 HFIP:EtOH (0.05 mg/mL), (**d**) 1:49 HFIP:EtOH (0.1 mg/mL), (**e**) 1:4 HFIP:EtOH (1 mg/mL), and (**f**) 1:9 HFIP:MeOH (0.5 mg/mL) mixtures.

Regarding to the Fmoc-FFFF-OFm tetrapeptide, the only reproducible assembly formed at room temperature from HFIP:water peptide solutions consisted on aggregates of nanospheres (Figure 17a), which were systematically observed for moderate peptide concentrations. On the other hand, stacked braids morphologies at the edges of the coverslips were found for 1:99 to 1:9 HFIP:EtOH solutions at 4 °C and Fmoc-FFFF-OFm concentrations ranging from 0.05 to 0.5 mg/mL (Figure 17b,c). As shown, the size of the braids, which was of around 13 µm in length and 3 μm in width, was almost independent of the peptide concentration. On the other hand, Fmoc-FFFF-OFm assembles into stable dendritic birefringent microstructures, which appear at room temperature from HFIP:EtOH solutions with peptide concentrations ranging between 0.5 and 2 mg/mL (Figure 17d,e) [57]. Similar branched structures are obtained when the EtOH co-solvent is replaced by MeOH, as shown in Figure 17f. The tendency of Fmoc-FFFF-OFm to form hierarchical dendritic microstructures in a relatively wide variety of conditions suggests that this tetrapeptide should be considered as a promising material for applications requiring ordered branched tree-like organizations.

On the other hand, comparison between Fmoc-FFFF and Fmoc-FFFF-OFm reveals that they organize forming completely different morphologies, with exception of the poorly defined nanospherical aggregates that are formed from dilute HFIP:water mixtures of the two peptides (Figure 10a and Figure 17a). Similarly, there is no trace of the formation of the fibrous structures characteristic of FFFF when the two ends of the peptide are capped with highly aromatic groups. In contrast, no other Phe-homopeptide has such a great tendency to form dendritic morphologies as Fmoc-FFFF-OFm, which illustrates that the tuning in the morphology of these materials is more drastic the higher the number of Phe residues.

Comparison of the self-assembly tendencies of Phe-homopeptides capped with fluorenyl groups at both the *N*- and C-terminus, which are schematically depicted in Figure 7b, with those of the corresponding uncapped peptides (Figure 7a) reveals significant differences. While the assembly preferences of uncapped peptides depend on whether the number of residues is even or odd, the microstructures formed by doubly capped peptides are related with the amount of π–π stacking interactions, which obviously increases with the number of Phe residues. Indeed, theoretical calculations indicated that Phe-homopeptides capped with fluorenyl groups at both the *N*- and C-terminus adopt antiparallel β-sheet assemblies rather than parallel β-sheets as their uncapped analogues, which was confirmed by FTIR spectroscopy [57]. Moreover, DFT calculations on highly aromatic Phe-homopeptides showed that the stability of the antiparallel β-sheet with respect to the parallel one increases with the number of Phe residues.

The assembly of uncapped Phe-homopeptides is dominated by –NH_3_^+^···^−^OOC– head-to-tail electrostatic interactions, favoring tubular, and fibrillar nano- and microstructures [33,39]. However, the stability of these structures is perturbed when the number of Phe changes from odd to even. This change in the interaction pattern results in the apparition of wide variety of morphologies, as for example helical ribbons, individual and grouped microplates, nanoplates and toroidal structures [39]. Instead, the assembly of Phe-homopeptides capped at the *N*- and C-terminus with hydrophobic fluorenyl groups evolves with the number of residues. More specifically, the microstructures formed by such peptides are stabilized by π–π stacking interactions, which are not only weaker but also more specific than head-to-tail electrostatic interactions. Because of this reason, the self-assembly of doubly capped homopeptides is very sensible to the peptide concentration and the length. Consequently, the self-assembly of doubly capped homopeptides progressively evolves with the increasing number of Phe residues, regardless of whether the number is odd or even.

## 7. Conclusions

The morphology of the self-assembled structures obtained from solutions of Phe-homopeptides without end-capping groups, capped at the *N*- or C-terminus with a fluorenyl group, and capped at the two termini with fluorenyl groups, has been reviewed. For this purpose, in addition of the peptide length, the following variables have been considered: the polarity and volatility of the co-solvent, the peptide concentration and, in some cases, the solvent and the temperature. The preferences of homopeptides with an even number of Phe residues towards fibrillar and tubular structures, in which molecules organize in a hexagonal (FF) or pseudo-hexagonal (FFFF) assemblies stabilized by electrostatic head-to-tail interactions, are altered when such interactions are eliminated and progressively substituted by π–π stacking interactions. A rich variety of morphologies have been identified for highly aromatic Phe-homopeptides, which have been attributed to the formation of hydrophobic cores formed by stacked aromatic rings. This versatility, combined with the design rules provided above for short Phe-homopeptides, can be used to fabricate of peptide materials with precise morphology, which cannot be achieved using peptides comprised of amino acids other than Phe. Therefore, morphological engineering based on the use of short Phe-homopeptides can be a powerful tool in the realization of promising functional materials for applications in many fields, such as biomedicine and photonics.
